# Anthelmintic-resistant nematodes in Irish commercial sheep flocks- the state of play

**DOI:** 10.1186/2046-0481-65-21

**Published:** 2012-12-22

**Authors:** Barbara Good, James Patrick Hanrahan, Daniel Theodorus de Waal, Andrew Kinsella, Ciaran Oliver Lynch

**Affiliations:** 1 Animal & Grassland Research and Innovation Centre, Teagasc, Co. Galway, Athenry, Ireland; 2 MedicineUCD Veterinary Sciences Centre, University College Dublin, School of Veterinary, Belfield, Dublin 4, Ireland; 3 UCD Veterinary Sciences Centre, Walsh fellow student previously based at University College Dublin, School of Veterinary Medicine, Belfield, Dublin 4, Ireland; 4 Teagasc, Coolruss, Tinahely Co.Wicklow, Ireland

**Keywords:** Anthelmintic resistance, Nematodes, Sheep Ireland, Benzimidazole, Levamizole, Ivermectin, MALDT, FECRT

## Abstract

Anthelmintic resistance has been reported in most sheep producing countries. Prior to the mid 1990s, reports of anthelmintic resistance in Ireland were sparse and focused on benzimidazole, one of the three classes of anthelmintic available during this period. This evidence for efficacy issues on Irish farms combined with awareness that anthelmintic resistance was increasingly being reported in other countries prompted the need for more comprehensive investigations on Irish farms. Faecal egg count reduction and micro-agar larval development tests were employed to investigate resistance to benzimidazole, levamisole and macrocyclic lactone. There is compelling evidence for resistance to both benzimidazole (>88% of flocks) and levamisole (>39% of flocks). Resistance of nematode populations to macrocyclic lactone was suspected on a small number of farms (11%) but needs to be confirmed. The recent introduction of two new classes of anthelmintics, after over a 25 year interval, together with the evidence that anthelmintic resistance is reported within a relatively short time following the introduction of a new anthelmintic compound means that the challenge to the industry is immediate. Actions are urgently required to manage anthelmintic resistance so as to prolong the lifespan of anthelmintics.

## Background

The effective management of the effects of gastrointestinal nematode parasites on grazing livestock relies heavily on the use of highly efficacious chemotherapy. With the recent introduction of two new classes of anthelmintic (amino-acetonitrile derivatives (AADs) in 2010 and spiroindole (SI) in 2012) to the Irish market, there are now five anthelmintic classes available for the control of gastrointestinal nematodes of sheep. Anthelmintic resistance (AR) has been identified in most sheep producing countries worldwide [1]. The evidence for nematode resistance to anthelmintics, in particular to the benzimidazoles, is overwhelming. Furthermore, there are increasing reports of multi-drug resistant nematodes [2]. The first evidence for AR in nematode populations on Irish sheep farms was reported in the early 1990s [3-5].

The results of a postal survey of Irish sheep producers suggest that many producers lack an awareness of the principles that underpin the sustainable use of anthelmintics and the practices that preserve anthelmintic efficacy [6]. Moreover, testing for resistance was rarely practiced. Failure to identify AR and manage its development will incur severe production penalties due to the impact of parasitic gastroenteritis. Once resistance to a particular class of anthelmintic emerges in a nematode population then parasitic gastroenteritis can no longer be controlled using that anthelmintic group.

The most commonly used methods for detecting anthelmintic resistance are the faecal egg count reduction test (FECRT) and the micro-agar larval development test (MALDT), both of which can be used to detect resistance to benzimidazole (BZ) and levamisole (LM). The FECRT involves calculating the mean reduction in faecal egg count (FEC) at a defined interval post-treatment for a sample of animals from a flock. The MALDT is based on the development of larvae (from eggs in a pooled fresh faecal material from a sample of animals in a flock) in various concentrations of the anthelmintic. A commercially available MALDT is also available DrenchRite®, (Microbial Screening Technologies, New South Wales, Australia) [7] with capability for checking resistance to BZ, LM, combination drugs (BZ + LM) and two macrocylic lactone (ML) analogues (avermectin and milbemycin). The findings from AR studies carried out on Irish commercial flocks between 2002 and 2010, using a range of tests available to assess AR, are summarised in this communication.

## Methods

Information on anthelmintic resistance was obtained using the following tests: FECRT and two *in vitro* MALDT tests, namely, a non-commercial MALDT and a commercial MALDT (DrenchRite®). For each of the studies, the farms involved were lowland farms each with a long-established sheep enterprise and a flock size greater than 100 ewes. Thus, they can be considered to represent the most committed participants in lowland sheep production.

The FECRT method was used to assess AR in flocks in three separate studies (the first FECRT study was undertaken in 2002 and included 11 farms (Co. Wicklow n = 7, Co. Monaghan n = 4) that were part of a collaborative project with Teagasc [8,9]. The second study, undertaken in 2006, involved 7 farms (Co. Kilkenny) belonging to members of a sheep producer discussion group [10]. The third, and most recent, study was undertaken in 2010, and included 3 farms that were participants in the Teagasc BETTER (Business, Environment and Technology through Extension and Research) farm programme [11,12]. In all of these studies, the farmers were fully briefed on the procedures required for the test and, on the initial visit, 30 lambs were chosen at random and marked to ensure unique identification at the subsequent visit. Individual faecal samples were taken *per rectum*, placed in air-tight bags and stored in a cooler box until refrigeration. Immediately post-sampling, 15 lambs were administered a BZ product (Systamex, Schering Plough Animal Health Ireland) and the other 15 lambs were given a LM product (Nilverm, Schering Plough Ltd in 2002, Nilzan Drench Plus, Schering Plough Ltd in 2006, 2010). Dosing procedures were, in all cases, according to manufacturer’s recommendations. Between days 10 and 14 post-treatment these lambs were resampled. The first two studies all took place during the autumn period and involved ewe lamb replacements. The third study took place in summer during the immediate post-weaning period. Only lambs with a pre-treatment FEC ≥100 eggs per gram (e.p.g.) where included in the actual analyses.

FECs were performed according to the modified McMaster methodology [13] and the arithmetic mean for each group of lambs was calculated pre- and post- treatment. The criteria used to evaluate anthelmintic resistance were based on the WAAVP recommendations [14]. Based on these criteria, resistance is present when the FEC reduction post-treatment is less than 95% and the lower limit of the 95% confidence interval for the percentage reduction is less than 90%. Resistance is ‘suspected’ when only one of these criteria is satisfied [14].

Participants in a nationwide survey of AR, using the non-commercial MALDT test, were sought from 108 respondents to a postal survey [6] concerning practices related to anthelmintic usage who had expressed an interest in providing samples for testing. Faecal sampling kits with instructions for flock sampling were subsequently forwarded to these respondents [15]. All faecal samples were processed within 48 h of postal delivery to recover nematode eggs for use in the MALDT. Sampling took place during the summer. The development of trichostrongyle eggs (excluding *Nematodirus* species) to third stage larvae (L3), after incubation at 25°C for 7 days, was examined over a range of concentrations of two anthelmintic drug classes, namely, BZ (thiabendazole) and LM, based on methods described by [16]. Controls were included in each assay (i.e. eggs not exposed to anthelmintic).

The rate of L3 development in the discriminating dose (0.02 μg/ml and 0.5 μg/ml for thiabendazole and levamisole, respectively) compared to the control was used to determine if resistance was present. [16]. L3 larvae from the control and those that developed in the discriminating anthelmintic dose were identified to species level. Keys as described by Soulsby [17] and Anon [13] were used as reference for identification.

In order to get an indication of the resistance to macrocyclic lactone a commercially available MALDT (DrenchRite®, Larval Development Assay; Microbial Screening Technologies, New South Wales, Australia) [7] was used in 2005 on a subset of the farms investigated in 2004. This included 5 farms where resistance to BZ and LM, based on the non-commercial MALDT, was indicated and 20 farms selected at random. The manufacturer’s instructions were followed in performing and interpreting the test. In this test nematode eggs were incubated in serial dilutions of BZ, LM, BZ + LM combination and ML.

All procedures described in this experiment were conducted under experimental license from the Irish Department of Health in accordance with the Cruelty to animal Act 1876 and the European Communities (amendments of the Cruelty to Animal Act 1976) Regulations 1994.

## Results

Three and 4 farms were excluded from the LM and BZ FECRT studies, respectively, because the average pre-treatment FEC was below 250 eggs per gram [14]. Resistance to BZ, based on FECRT, was evident in 15 flocks (88%) and to LM in 7 flocks (39%). Suspect resistance to BZ was evident in 1 flock (6%) and to LM, in 2 flocks (11%) (Table 1).

**Table 1:** Results of the Faecal egg count reduction tests on individual Irish flocks

**Year**	**Location†**	**Flock ID**	**Benzimidazole**				**Levamisole**			
			**Lambs**^‡^**(n)**	FECpre*	**%^**	**AR status**^**§**^	**Lambs (n)**	**FECpre**	**%**	**AR Status**
2002	WW	01	12	6502	88.3	R	12	5114	99.1	S
	WW	04	15	3250	72.8	R	10	1684	94.6	SR
	WW	05	16	1376	72.2	R	11	762	92.7	R
	WW	06	16	1598	56.0	R	10	441	100.0	S
	WW	07	12	3898	76.4	R	12	2411	98.8	S
	WW	08	14	1376	62.3	R	12	537	98.2	S
	MN	02	12	1571	94.1	SR	12	1630	89.6	R
	MN	04	10	1508	34.1	R	10	1205	91.5	R
	MN	06	13	1302	94.6	S	13	1344	97.2	S
2006	KK	01	11	336	23.7	R	10	585	99.1	S
	KK	02	14	377	24.7	R	14	365	98.1	S
	KK	03	13	1254	42.2	R	13	1092	89.0	R
	KK	04	10	1800	32.7	R	10	1790	81.0	R
	KK	05	12	745	56.8	R	12	933	97.1	S
	KK	06	11	523	32.6	R	11	536	83.7	R
	KK	07	14	1015	73.6	R	14	1154	96.3	SR
2010	KY	01	12	290	61.0	R	20	328	97.0	S
	WW**	02	12	150	9.1	-	7	314	85.2	R

^†^ WW = Co. Wicklow, Mn = Co.Monaghan, KK = Kilkenny, KY = Co.Kerry.

^‡^ number of lambs included in the final analysis.

^*^FECpre = Faecal egg count pre-treatment.

^**^**^Percentage reduction in faecal egg count observed post-treatment.

^**§**^ R = significant resistance (P < 0.05), SR = suspected resistance, S = susceptible to anthelmintic.

** results for BZ FECRT not included in statistical analysis as FEC_pre_ was ≤250 e.p.g.

**Figure 1:**
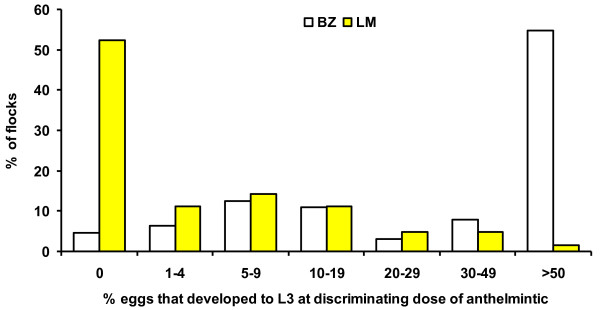
Flocks (BZ, n=64, LM, n=63) classified (%) according to the percentage of eggs that developed to third stage (infective) larvae in the discriminating concentration of thiabendazole (BZ) (0.02 μg/ml) or levamisole (LM) (0.5 μg/ml).

**Figure 2:**
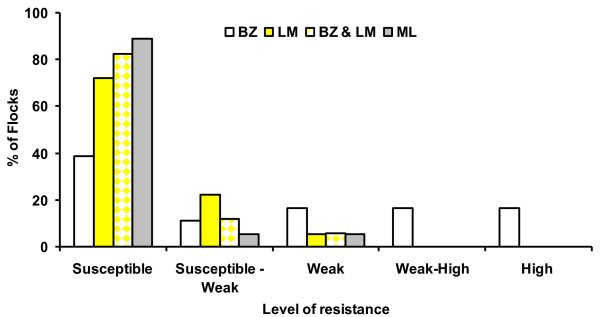
Incidence of resistance to benzimidazole (BZ) levamisole (LM), benzimidazole plus levamisole (BZ & LM)and macrocyclic lactone (ML) determined using the DrenchRite® assay.

Seventy-four samples were received for the MALDT test of which 64 and 63 were suitable for inclusion in the test for BZ and LM resistance, respectively. The number of farms and the percentage of eggs that developed to L3 in the discriminating dose are summarised in Figure 1 for BZ and LM. Evidence for resistance to BZ was observed in 95% (n = 61) of flocks while resistance to LM was evident in 48% (n = 30). *Teladorsagia* spp (formerly *Ostertagia*), *Trichostrongylus* spp and *Cooperia* spp. were the main species identified. Eighteen farms were suitable for inclusion in the DrenchRite® Assay; the results from this test (Figure 2) indicated that susceptibility to BZ and LM in nematode populations was observed on 39% and 72% of farms. Susceptibility to both BZ plus LM was observed in the nematode populations on 82% of farms while susceptibility to ML was observed in 89%.

## Discussion and conclusions

Similar incidences of resistance were observed across the studies regardless of the non-commercial test procedure employed. It is not immediately clear why the incidences from the commercial MALDT were lower and further work would be required to directly compare methods. It is noted that in the assessment of LM resistance using FECRT, the maturation of immature nematode stages may lead to false positive results when faecal egg counts are taken 11 or more days post treatment [18]. However, there was no evidence for any association between time (day 11 *v*. days 12–14) and the declaration of LM resistance (data not shown). Overall, the results clearly demonstrate the presence of multiple drug resistance in trichostrongyle worm populations on Irish farms. The evidence on the incidence resistance to BZ and LM is alarming. While *in vitro* methods for detecting ML resistance are being developed, FECRT remains the gold standard. Consequently, confirmation of the resistance to ML indicated by the DrenchRite® assay is required. With increasing reports of resistance to ML elsewhere [2] combined with the evidence that AR emerges within a relatively short time period (<10 years) following the commercial release of a new anthelmintic compound for sheep [1,19] mean that the challenge facing Irish sheep producers is of serious concern, and requires immediate actions to increase awareness and understanding of procedures necessary to minimise the risk of emergence of resistance.

It is important that the current situation, where incidence of anthelmintic resistance is high or very high for two of the available anthelmintic classes, is recognised as a direct consequence of past actions (such as suppressive treatment and under dosing) that inadvertently selected for resistant parasites. These and other sub-optimal practices were identified in the survey on parasite control practices on lowland sheep [6]. In light of the recent introduction of two new anthelmintic classes, it is imperative that producers and the wider industry are proactive in managing anthelmintic resistance on farms so that past mistakes are avoided. The recommendations, as set out by the SCOPs (Sustainable Control of Parasites) working group, to delay the emergence of AR and prolong the lifespan of remaining and any new anthelmintic classes are pertinent. The effective administration of appropriate anthelmintics in a targeted manner as well as the adoption of bio-security practices and AR testing are among the key guidelines. In addition, novel strategies to preserve susceptible worms on farms and reduce the frequency of anthelmintic usage are described [20].

The “Food Harvest 2020 A vision for Irish Agri-food and Fisheries ” report calls for a growth of 20% in the value of sheep output by 2020 [21]. The existence of high levels of resistance to some anthelmintic families, the presence of farms with multiple drug resistance, and the potential that the incidence of resistance to ML and more recently introduced anthelmintics will increase pose a serious threat to the achievement of this goal by the Irish sheep sector.

## Abbreviations

AR: Anthelmintic resistance; BETTER: Business, environemnt and technology through extension and research; BZ: Benzimidazole; Epg: Eggs per gram; FEC: Faecal egg count; FECRT: Faecal egg count reduction test; IVM: Ivermectin; LDT: Larval development test; LM: Levamisole; MALDT: M larval development test; TET: Technology evaluation transfer.

## Competing interests

The authors’ declare that they have no competing interests.

## Authors’ contributions

BG JPH and TDW conceived the studies. BG and JPH participated in the design and coordination of all AR studies. BG performed statistical analysis of results and prepared the manuscript. JPH provided advice on design, statistical analysis for all AR studies and preparation of the manuscript. TDW participated in the design, co-ordination and analysis of data from the MALDT and participated in the writing of the manuscript. TP participated in the coordination and completion of laboratory based MALDT. AK coordinated and collected samples from the TET farms in 2003. COL participated in design, co-ordination and collection of samples from the BETTER farms in 2010. All authors read and approved the final manuscript.

## Authors’ information

BG B.A.Mod., Dip stat, PhD, Parasitologist Senior research Officer in Teagasc, JPH, B.Agr.Sc. PhD, former Senior Principal Research Officer and Head of Sheep Research Centre, Teagasc, Athenry now retired, TDW, BVSc, PhD, DipDatMet, HDipUTL, DipEVPC, MRCVS, Senior Lecturer and European Veterinary Specialist in Parasitology in UCD, AK, B.Agr.Sc former Sheep specialist advisor, Teagasc, now retired, TP MSc. Walsh fellow student, Teagasc Masters awarded in 2005, COL B.Agr.Sc. Technologist,Teagasc.
